# Beyond borders: A case report of small bowel obstruction secondary to undiagnosed florid endometriosis

**DOI:** 10.1016/j.ijscr.2022.106994

**Published:** 2022-03-29

**Authors:** Amenah Dhannoon, Amrit Bajwa, Mobarak Kunna, Aoife Canney, Emmeline Nugent

**Affiliations:** a Royal College of Surgeons in Ireland, 123 St Stephen's Green, Dublin, Ireland; b Department of Surgery, University Hospital Galway, Galway, Ireland; c Department of Pathology, University Hospital Galway, Galway, Ireland; d Academic Department of Surgery, National University of Ireland Galway, Galway, Ireland

**Keywords:** Endometriosis, Terminal ileum, Bowel obstruction, Case report

## Abstract

**Introduction and importance:**

Endometriosis is a gynecological condition referring to the presence of endometrial tissue outside the endometrium with the potential of progressing to malignancy. It mostly affects pelvic organs; however, it has been described beyond the pelvis. In 10% of cases it occurs in the bowel, mostly rectum and sigmoid. Involvement of the small bowel is rare. Here we report endometriosis of the terminal ileum and appendix in a patient with no previous diagnosis of endometriosis.

**Case presentation:**

We describe a case of a 39-year-old-female who presented with abdominal pain, nausea and vomiting to the emergency department. This was on background history of intermittent abdominal pain every 2 weeks for the previous 5 months. Further investigation with computed tomography (CT) of the abdomen and pelvis showed small bowel dilatation with a polypoidal lesion obstructing the terminal ileum. On colonoscopy, no intraluminal lesions were identified in the terminal ileum. The patient underwent right hemicoloectomy. Histopathological results revealed endometriosis. The patient had uneventful recovery post-operatively and at her follow-up review at 4 weeks and 2 months from surgery.

**Discussion:**

The presentation of endometriosis of the bowel is highly variable and difficult to diagnose pre-operatively. Due to lack of specific diagnostic measures, surgical resection and histology can be the only reliable way for first-time endometriosis diagnosis presenting as small bowel obstruction.

**Conclusion:**

Extra-pelvic endometriosis should be considered as the cause of small bowel obstruction in the absence of other causes of bowel obstruction in young female patients.

## Highlights

• Bowel endometriosis can be asymptomatic or can present with wide range of symptoms
• Small bowel obstruction is a rare presentation of first-time endometriosis diagnosis
• There is no specific diagnostic measure for endometriosis presenting as small bowel obstruction
• Intra-operative and histological examinations can be the only reliable measure to diagnose small bowel endometriosis.

## 1 Introduction

Endometriosis is a benign gynecological disease defined as the presence of normal endometrial mucosa that is abnormally present outside the uterine cavity. It affects approximately 5–15% women of reproductive age [1]. It most commonly involves the pelvic organs but it has been observed in extra-pelvic organs such as the intestine, bladder, abdominal wall and thoracic cavity [2].

Endometriosis of the bowel accounts for 10% of cases with the majority involving the rectum and sigmoid (80–90%). It typically presents as a single nodule, with a diameter greater than 1 cm, commonly infiltrating the muscularis layer of the bowel and the surrounding structures [1], [2], [3].

Clinical symptoms, examination, biological tests and imaging modalities are all non-specific in diagnosing endometriosis and therefore careful consideration should be given to rule out endometriosis in young female patients with chronic abdominal symptoms [1], [2], [3]. Clinical presentation of endometriosis is highly variable according to the organ affected. The most common presentation when terminal ileum/appendix is affected is bowel obstruction, perforation, acute appendicitis and intussusception [3].

Medical management is widely used as the first step in the treatment of pelvic endometriosis, with surgical intervention becoming indicated after failure of medical management. Nevertheless, in female patients with no previous confirmed diagnosis, surgical intervention can be both diagnostic and therapeutic [1].

In this case report, we describe a case of terminal ileum endometriosis presenting as small bowel obstruction. This report serves as a reminder that endometriosis should be considered as a potential differential in a young, healthy female in her reproductive years presenting with bowel obstruction.

## 2 Case report

A 39-year-old Hispanic lady presented to the emergency department with a one-day history of severe colicky non-radiating lower abdominal pain with nausea, several episodes of vomiting and constipation. She reported 3 kg of unintentional weight loss over the previous five months. She reported a five-month history of undiagnosed intermittent abdominal pain. In previous episodes, the pain recurred every two weeks and settled spontaneously. On this occasion the pain did not resolve. She had no past medical or surgical history. Her menstrual cycle was regular with no menorrhagia or dysmenorrhea. She was not any regular medications and there was no contributory family history. She had a 7.5 pack year smoking history with occasional alcohol consumption.

On presentation, she was haemodynamically stable. Physical examination revealed abdominal distension with tenderness in the lower abdomen. Digital rectal examination was performed and revealed an empty rectum. Her biochemical and haematological tests including tumor markers were within normal limits and her urine dipstick was normal.

**Fig. 1:**
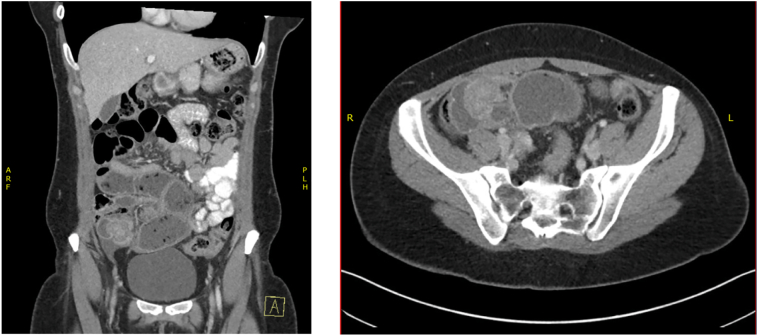
A (coronal view) and B (axial view) of CT abdomen/pelvis demonstrating small bowel dilation and polypoid lesions as highlighted by blue arrows.

The patient had plain film abdomen which showed multiple air-fluid levels associated with dilated bowel loops with no air in the rectum. Subsequently, she underwent a computed tomography (CT) of the abdomen and pelvis which demonstrated a focal area of small bowel dilation in the distal ileum with a polypoid lesion measuring 3.4 cm × 3.1 cm × 2.8 cm (Fig. 1). Free fluid adjacent to the caecum and prominent ileocolic lymph nodes were also noted. The large bowel, ovaries and uterus were unremarkable. She proceeded to colonoscopy to evaluate the terminal ileal polypoid lesion identified at CT. Colonoscopy did not demonstrate an intra-luminal lesion.

**Fig. 2:**
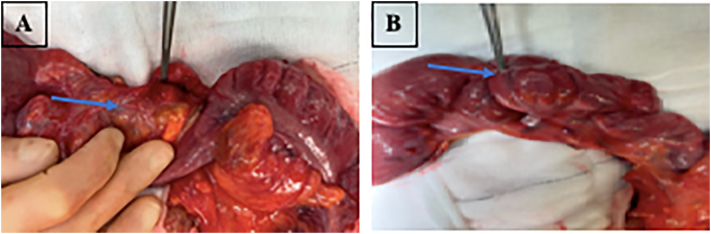
A and B; specimen with polypoid mass attached to the serosa of the terminal ileum as highlighted by blue arrows.

As her symptoms persisted and given the CT findings the patient proceeded to diagnostic laparoscopy performed by a consultant colorectal surgeon. This revealed a right ovarian haemorrhagic cyst and prominent dilated small bowel. There was also a transition point noted in the distal ileum with the appendix tethered to the ileum forming a mass (Fig. 2). Given these intra-operative findings a right hemicolectomy with end to side anastomosis was performed.

**Fig. 3:**
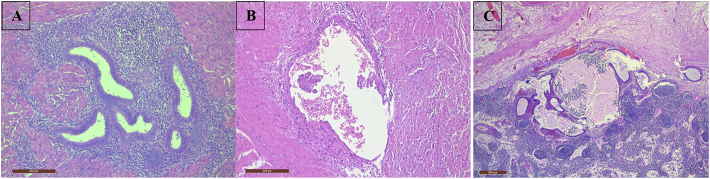
A; High power view of benign endometrial glands and stroma within smooth muscle of the terminal ileum muscularis propria. No atypia present. B; Focus of endometriosis with endometrial cells demonstrating possible papillary architecture with mild cytological atypia. C; Mesenteric lymph node involved by endometriosis. There is no evidence of malignancy.

The microscopic examination of the specimen confirmed benign endometrial glands and stroma within smooth muscle of the terminal ileum muscularis propria. No cell atypia was identified. Mesenteric lymph node was involved by endometriosis without evidence of malignancy (Fig. 3).

The patient was referred to the gynaecology department for further management of endometriosis. She underwent Magnetic Resonance Imaging (MRI) of the pelvis which was unremarkable. The patient made an uneventful recovery and was discharged home on day 13. She continued to do well with return to her normal daily activities at her 4-week and 2-month clinic review. She declined treatment for her underlying endometriosis.

## 3 Discussion

Endometriosis is a chronic inflammatory, estrogen-dependent condition that can be divided into genital versus extra-genital with the bowel being the most common location for extra-genital endometriosis [1], [2], [3].

The most common symptoms leading to a diagnosis of endometriosis are dysmenorrhea (79%), pelvic pain (69%), and subfertility (53%), none of which were present in our case [1]. Although bowel endometriosis can be entirely asymptomatic, it can present with a wide range of symptoms including, nausea, vomiting, colicky abdominal pain, rectal pain, mass formation and rarely intestinal obstruction [5]. It also commonly presents as a single nodule, with a diameter of >1 cm, with infiltration of the muscularis propria and surrounding structures, with 0.15% of patients presenting with obstruction as seen in our case [1], [6], [7]. When an endometrial lesion invades the retro-peritoneal wall or the wall of an organ a depth greater than 5 mm, it is referred to deep infiltrative endometriosis (DIE) [9]. Deep infiltrative endometriosis has been further classified by Remorgida et al. (Table 1). This was in an effort to investigate the relationship between GI symptoms and histological findings. They concluded that all women presenting with stage 1–3 bowel endometriosis reported bowel complaints. In our patient the endometriosis was seen as deep as the muscularis propria and therefore stage 2.

**Table 1:** Staging of bowel endometriosis and prevalence according to 2005 study conducted by Remorgida et al. [8].

Stage	Depth of infiltration	# of cases
0	Lesions confined to serosal layer and surrounding connective tissue	45/68
1	Lesions infiltrated subserous plexus	11/68
2	Deep infiltration of muscular wall and disruption of the Auerbach plexus	8/68
3	Infiltration reached submucosal Meissener plexus or the mucosa itself	4/68

Although isolated bowel endometriosis has been previously reported, many patients will have a previous history of endometriosis [10]. It is also important to note that the average lag time from onset of symptoms to achieving a diagnosis of endometriosis is 6 years or up to 12 in the United States [11]. In women that have undiagnosed endometriosis, physical examination can be of limited use with little sensitivity and specificity and many cases would be diagnosed post-surgically [1], [2], [3]. Intestinal endometriosis is therefore commonly misdiagnosed as irritable bowel syndrome (IBS), acute appendicitis or diverticulitis, inflammatory bowel disease, intestinal carcinoma, or ovarian pathology [12], [13]. Unfortunately however, there are no non-invasive tests to diagnose or differentiate between IBS and bowel endometriosis, In case report by Soumekh, the patient had a 4-year history of presumed IBS that was only later diagnosed as small bowel endometriosis [4].

Non-invasive imaging has limited use in the diagnosis of endometriosis especially when taking into consideration the large range of potential sites of DIE. A network meta-analysis and systematic review conducted by Hudelist et al. in 2011 suggested that transvaginal sonography (TVS) is a valuable tool for the non-invasive diagnosis of recto-sigmoid endometriosis [14]. TVS is low cost, readily available, but is limited in its ability to detect infiltration of the mucosal layer and has high user dependency [6]. Further, despite the recto-sigmoid being the most common location of DIE, this would have been ineffective in diagnosing our patient. Both TVS and TRS have limited use for the surgeon when determining the most appropriate surgical resection. MRI is recommended as the second investigation for DIE for its high sensitivity of 88% and specificity of 98% coupled with a diagnostic accuracy of 96% [6]. Double contrast barium enema and CT colonography are also proposed modalities to aid in surgical treatment [6].

Knowledge of pre-existing endometriosis as well as varying clinical presentations play a key role when choosing the most appropriate imaging modality for further investigation. Despite these imaging modalities available, bowel endometriosis is often an incidental finding during surgical treatment as in our case. Ileal endometrioma's however can mimic malignancy by invading the small bowel lumen and further invasive modalities such as colonoscopy can be used to out rule malignancy [15]. In our case report however, no lesions were identified as the incidence of colonoscopy findings in DIE are as low as 4% [16].

The choice of surgical technique depends most importantly on location of the bowel lesion and further characteristics of lesion such as size and number of nodules and depth of infiltration [17]. With a high clinical suspicion for malignancy, segmental resection and primary anastomosis was the choice of procedure for this patient. This technique allows for a radical removal of intestinal endometriosis and minimizes future risk of recurrences, improves pelvic pain, intestinal symptoms and quality of life [17].

Medical therapies have also been shown to be effective in the long-term management of endometriosis with progestins most used. Few studies however have specifically investigated the use of hormonal therapies in bowel endometriosis. A prospective study has shown that norethisterone acetate and gonadotrophin-releasing hormone agonists (GnRH-a) have success in selected cases but are limited by side effects and recurrence on cessation of the medication [18]. Evidently, these would not be suitable options in a patient presenting with acute bowel obstruction.

In conclusion, this case highlighted the first presentation of endometriosis as small bowel obstruction. Endometriosis should be therefore considered as a rare cause of small bowel obstruction in the absence of conclusive radiological and endoscopic imaging in female patients of childbearing age.

The case has been reported in line with the SCARE 2020 criteria [19].

## Patient consent

Patient written consent has been obtained to publish this case.

## Consent

Written informed consent was obtained from the patient for publication of this case report and accompanying images. A copy of the written consent is available for review by the Editor-in-Chief of this journal on request.

## Provenance and peer review

Not commissioned, externally peer-reviewed.

## Ethical approval

None.

## Funding

None.

## Guarantor

Amenah Dhannoon.

## Research registration number

None.

## CRediT authorship contribution statement

Amenah Dhannoon: Writing- Original draft preparation, resources, visualisation
Amrit Bawja: Draft writing
Mobarak Kunna: Conceptualization
Aoife Canney: Resources
Emmeline Nugent: Operating surgeon, supervision.

## Declaration of competing interest

None.
